# The use of thermal imaging for evaluation of peripheral tissue perfusion in surgical patients with septic shock

**DOI:** 10.1186/s12871-024-02486-w

**Published:** 2024-03-21

**Authors:** Ahmed Hasanin, Radwa Fekry, Maha Mostafa, Sahar Kasem, Amany Eissa, Hassan Mohamed, Heba Raafat

**Affiliations:** Department of Anesthesia and Critical Care Medicine, Cairo University, Cairo, Egypt

**Keywords:** Infrared thermal imaging, Temperature, Septic shock, Lactate, Capillary refill time, Peripheral perfusion, Mortality, Emergency surgery

## Abstract

**Background:**

In this study, we aimed to evaluate the ability of central-to-peripheral temperature gradients using thermal imaging to predict in-hospital mortality in surgical patients with septic shock.

**Methods:**

This prospective observational study included adult patients with septic shock admitted to the intensive care unit postoperatively. Serum lactate (in mmol/L), capillary refill time (CRT) (in seconds), toe (peripheral) and canthal (central) temperature by infrared thermography and the corresponding room temperature in (Celsius [°C]) were assessed at the time of admission, 6- and 12 h after admission. The canthal-toe and room-toe temperature gradients were calculated. According to their final outcomes, patients were divided into survivors and non-survivors. The ability of canthal-toe temperature gradient (primary outcome), room-toe temperature gradient, toe temperature, serum lactate and CRT, measured at the prespecified timepoints to predict in-hospital mortality was analyzed using the area under receiver operating characteristic curve (AUC).

**Results:**

Fifty-six patients were included and were available for the final analysis and 41/56 (73%) patients died. The canthal-toe and room-toe temperature gradients did not show significant accuracy in predicting mortality at any timepoint. Only the toe temperature measurement at 12 h showed good ability in predicting in-hospital mortality with AUC (95% confidence interval) of 0.72 (0.58–0.84) and a negative predictive value of 70% at toe temperature of ≤ 25.5 °C. Both serum lactate and CRT showed good ability to predict in-hospital mortality at all timepoints with high positive predictive values (> 90%) at cut-off value of > 2.5–4.3 mmol/L for the serum lactate and > 3-4.2 s for the CRT.

**Conclusion:**

In post-operative emergency surgical patients with septic shock, high serum lactate and CRT can accurately predict in-hospital mortality and were superior to thermal imaging, especially in the positive predictive values. Toe temperature > 25.5 °C, measured using infrared thermal imaging can exclude in-hospital mortality with a negative predictive value of 70%.

## Introduction

Evaluation and maintenance of peripheral tissue perfusion is essential during management of septic shock [1, 2]. Classically, resuscitation of septic shock patients targets macro-circulatory parameters such as mean arterial pressure and cardiac output; however, it had been proved that alteration of microcirculatory perfusion indices is more predictive for patient outcomes than macro-circulatory indices [3]. There are several markers for peripheral perfusion and each has its specific pros and cons. Serum lactate is still the standard measure for tissue perfusion; however, it has several limitations and can sometimes be unavailable in resource limiting settings [4]. All other markers of tissue perfusion have limitations such as being invasive (e.g., central venous oxygen saturation), or time-lagging (e.g., urine output) [2].

There is an increased interest in the evaluation of non-vital organs (e.g., skin and skeletal muscles) as surrogates of microcirculation because non-vital organs deteriorate earlier and recover later than the vital organs [5]; among these organs the skin, that is frequently used for this purpose since it is easily accessible and provides rapid and simple assessment of peripheral perfusion. There are various indices for evaluation of skin perfusion such as capillary refill time (CRT), skin mottling, and temperature gradients. Temperature gradients have some advantages over other skin perfusion indices: 1- better reproducibility than the CRT. 2- more suitable in dark skin than skin mottling score [2]. Furthermore, a recent meta-analysis questioned the prognostic value of the CRT in adult patients at risk of death or acute circulatory failure [6]. Temperature gradients such as central-to-toe gradient and toe-to-ambient gradient were reported as perfect perfusion indices [7]. Temperature gradients are usually measured through special probes which are sometimes unavailable. Furthermore, the recent pandemic of the Coronavirus disease-2019 produced a serious collapse in the health resources in many countries; this breakdown raised the interest in the use of methods which: 1- can be used without close contact with the patient to avoid infection, 2- can be used in many patients without expensive disposables, and 3- can be used by non-medical personnel in case of shortage of trained physicians.

Thermal imaging is an evolving route for evaluation of skin temperature which showed various clinical benefits such as evaluation of nerve block success [8, 9], early detection of shock [10], and deep-tissue pressure injury [11]. Thermal imaging had also showed good ability for evaluation of peripheral perfusion in patients with acute limb ischemia [12]. Thermal imaging can provide an effective method for evaluation of peripheral perfusion, especially in cases of mass admissions and shortage of resources. In this study, we aimed to evaluate the ability of central-to-peripheral temperature gradient measured by infrared thermal imaging to predict in-hospital mortality in adult surgical patients with septic shock. We also aimed to assess the relation between temperature gradient and lactate as a marker for perfusion.

## Methods

### Ethics approval

This prospective observational study was conducted in trauma and surgical intensive care unit (ICU) of Cairo University Hospital, after the institutional ethical committee approval (MD-24-2021), from March 2021 to May 2022. Informed consent was obtained from the patient’s next-of-kin prior to enrollment into the study.

### Population

We included all consecutive adult (> 18 years) patients with clinically suspected septic shock according to the latest definition of septic shock (sepsis-3) [13] who were admitted to the ICU postoperatively. Patients with evident blood loss or metastatic cancer were excluded from the study. We also excluded patients with uncontrolled septic focus since these patients are at-risk of deterioration and poor prognosis. Patients with conditions that preclude the measurement of temperature from the toe and/or medial canthus such as infection, inflammation or ischemia were also excluded.

### Measurements

Perfusion indices including serum lactate (in mmol/L), CRT (in seconds) were assessed at the time of admission, 6- and 12 h after admission. The CRT was assessed by application of pressure on patient’s finger nailbed for nearly 5 s until its blanching then the pressure was released. The time for finger nailbed to regain its color was recorded.

### Temperature measurements

Using FLIR C2 compact thermal camera (FLIR Systems, Oregon, USA). The peripheral temperature was measured in Celsius (°C) at the toe and central temperature was measured at the medial canthus on admission, 6- and 12 h after admission. The thermal camera was placed 0.5 m from the designated areas. Scanning was conducted over a period of 10 s until the temperature reading became stable [14, 15]. An independent researcher recorded the temperature. The FLIR C2 thermal camera was calibrated before use. The corresponding room temperature was recorded by the same researcher (using Foonee Digital Hygrometer Indoor Thermometer, China).

The canthal-toe temperature gradient was calculated as the difference between the medial canthus and toe temperature. The room-toe temperature gradient was calculated as the difference between room and toe temperature.

Patients were followed during their ICU course. According to their final outcome, patients were divided into survivors and non-survivors.

### Outcomes

The primary outcome was the ability of canthal-toe temperature gradient at the time of ICU admission to predict in-hospital mortality.

Secondary outcomes included the ability of room-toe temperature gradient, toe temperature, serum lactate and CRT measured at the prespecified timepoints to predict in-hospital mortality.

Correlation between each of temperature gradient and toe temperature with serum lactate levels.

Upon admission to the ICU, the following data were collected: demographic data (age, sex, height, comorbidity), the acute physiology, and chronic health evaluation II (APACHE II) score, sequential organ failure assessment (SOFA) score (the scores were calculated and recorded without recording the included laboratory data), hemodynamic variables (mean arterial pressure, heart rate), vasopressor requirements and laboratory data (arterial blood gases and serum creatinine). Type of surgical intervention, days of mechanical ventilation and ICU stay were also recorded.

### Statistical analysis

We calculated the sample size using the MedCalc Software version 14 (MedCalc Software bvba, Ostend, Belgium) to detect the accuracy of temperature gradient in predicting patient mortality. The sample size was calculated to detect an area under receiver operating characteristics curve (AUC) of 0.8 and the null hypothesis was set at AUC of 0.5. A minimum number of 38 patients with at least 19 mortality cases was needed for a study power of 80% and an alpha error of 0.05.

The statistical package for social science (SPSS) for Microsoft version 26 (IBM Corp., Armonk, NY, USA) and MedCalc software were used for data analysis. Patients were divided into survivors and non-survivors. Categorical data are expressed as frequency (%). Normality of the numerical data was assessed using the Shapiro-Wilk test. Numerical data are expressed as means ± standard deviation, or medians (quartiles) as appropriate. Repeated measured data were analyzed using the analysis of variance test for repeated measures. The Bonferroni test was used for adjustment for multiple comparison. The AUC analysis was performed to evaluate the ability of temperature measurements, lactate, and CRT to predict in-hospital mortality. The best cut-off value was calculated using the Youden index and the corresponding positive and negative predictive values were reported. Comparison between the AUCs that showed statistical significance was done using the Delong test. The correlation between the temperature measurements and serum lactate values were done using the Spearman’s correlation coefficient. The level of significance was set at *P* < 0.05.

## Results

**Fig. 1:**
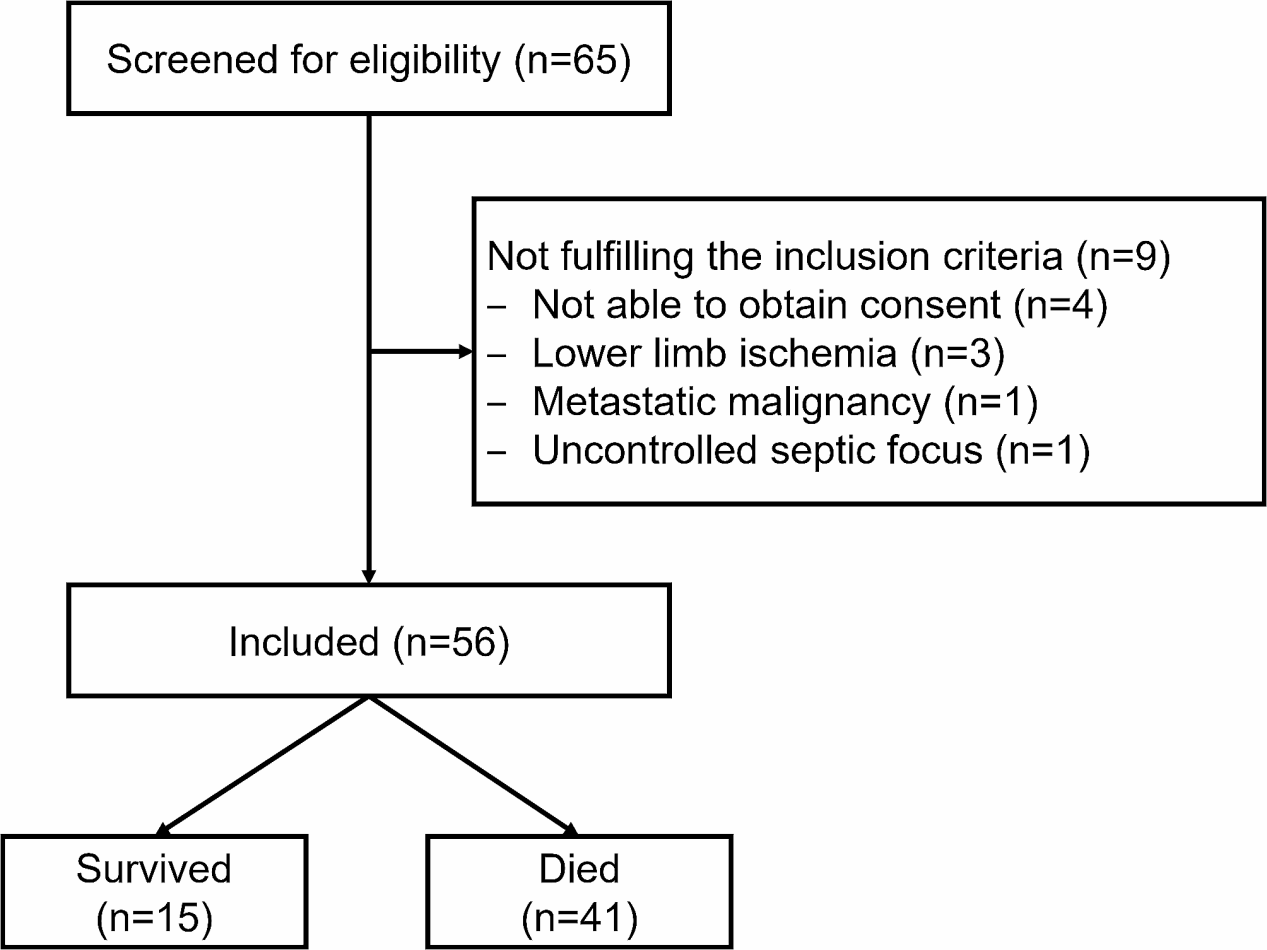
Patients’ enrollment

Sixty-five patients were screened for eligibility from whom 9 patients were excluded for not fulfilling the inclusion criteria. Fifty-six patients were included and were available for the final analysis. The median (quartiles) ICU stay was 7 (3, 11) days and 41/56 (73%) patients did not survive (Fig. 1).

The non-survivors had higher age, APACHE II score, SOFA score, baseline serum creatinine and lower mean arterial pressure, *p*H and HCO_3_ than the survivors (Table 1). Furthermore, the non-survivors required more vasopressor support than the survivors (Table 1). The type of surgery was similar between the two groups.

**Table 1:** Demographic data, vital signs, and laboratory data. Data are presented as mean ± standard deviation, median (quartiles) and frequency (%)

	Survivors (*n* = 15)	Non-survivors (*n* = 41)	*P*-value
Age (years)	46 ± 16	61 ± 12	< 0.001
Male sex (%)	8 (53%)	26 (63%)	0.494
Height (cm)	170 (160, 180)	170 (160, 175)	0.628
Comorbidity			
Hypertension Diabetes Meletus Ischemic heart disease Atrial fibrillation Liver dysfunction Chronic kidney disease	5 (33%) 7 (47%) 3 (20%) 15 (100%) 0 (0%) 1 (7%)	11 (27%) 14 (34%) 4 (10%) 38 (93%) 6 (15%) 2 (5%)	0.633 0.391 0.305 0.556 0.177 1.000
Type of surgery			
Small bowel resection Large bowel resection Liver abscess drainage Soft tissue debridement Amputation	7 (47%) 3 (20%) 0 (0%) 2 (13%) 3 (20%)	15 (37%) 5 (12%) 2 (5%) 11 (27%) 8 (20%)	0.552
Reoperation	5 (33%)	14 (34%)	0.955
APACHE II score	17 (14, 19)	20 (18, 25)	0.007
SOFA score	6 (5, 8)	8 (7, 10)	0.003
Baseline heart rate (bpm)	104 ± 25	101 ± 22	0.676
Baseline mean arterial pressure (mmHg)	97 ± 10	83 ± 13	0.001
No. of vasopressors at admission			0.009
1 2	15 (100%) 0 (0%)	27 (66%) 14 (34%)	
Noradrenalin dose (mcg/kg/min)	0.2 (0.1, 0.4)	0.4 (0.2, 0.6)	0.011
Adrenaline dose (mcg/kg/min)	0.0 (0.0, 0.0)	0.0 (0.0, 0.2)	0.011
*p*H	7.30 ± 0.09	7.21 ± 0.12	0.020
Baseline HCO_3_ (mmol/L)	18.8 (16.5, 21)	15.8 (11.3, 18.8)	0.020
Serum creatinine (mg/dL)	1.1 (0.8, 1.5)	2.0 (1.3, 3.4)	0.002
No. of patients who were mechanically ventilated	9 (60%)	41 (100%)	< 0.001
Days of mechanical ventilation	1 (0, 2)	4 (2, 10)	< 0.001

APACHE II: acute physiology, and chronic health evaluation II score, SOFA: sequential organ failure assessment score

The canthal-toe temperature gradient was higher in the non-survivors than survivors at 12 h after admission (*P*-value: 0.004). The toe temperature was lower in the non-survivors than the survivors at 6 and 12 h after admission (*P*-values: 0.036 and 0.001, respectively). On the other hand, there was no significant difference between the survivors and non-survivors regarding the room-toe temperature gradient (Table 2). Serum lactate and CRT were higher in non-survivors in relation to survivors at all time points (Table 2).

**Table 2:** Temperature measurements and perfusion indices. Data presented as mean ± standard deviation and median (quartiles)

	Survivors (*n* = 15)	Non-survivors (*n* = 41)	*P*-value
Canthal-toe temperature gradient (°C)			
0	12.8 ± 3.8	12.8 ± 3.8	0.986
6 h	11.2 ± 5.0	13.3 ± 3.4	0.091
12 h	9.9 ± 6.1	13.9 ± 3.7	0.004
Room-toe temperature gradient (°C)			
0	0.5 (-1.0, 2.5)	1.2 (-0.4, 2.8)	0.385
6 h	-0.7 (-0.4, 1.0)	0.1 (-2.1, 1.0)	0.401
12 h	-0.5 (-10.9, 1.7)	0.8 (-2.0, 2.5)	0.179
Toe temperature (°C)			
0	20.9 ± 3.8	20.9 ± 3.7	0.969
6 h	23.7 ± 4.8	21.1 ± 3.5	0.036
12 h	25.6 ± 6.4	20.8 ± 3.5	0.001
Serum lactate (mmol/L)			
0	2.5 (2.2, 4.2)	5 (2.7, 8.4)	0.011
6 h	2.4 (1.4, 3.8)	4.7 (3.1, 8.6)	0.001
12 h	1.9 (1.2, 2.5)	4.2 (2.3, 7.9)	< 0.001
CRT (s)			
0	2.5 (1.5, 3.0)	4.0 (3.0, 5.5)	0.006
6 h	2.0 (1.5, 2.5)	4.0 (2.8, 5.2)	0.001
12 h	1.5 (1.2, 3.0)	4.5 (1.8, 6.8)	< 0.001

CRT: capillary refill time

The canthal-toe and room-toe temperature gradients did not show significant accuracy in predicting mortality at any timepoint (*P*-value > 0.05) (Table 3). The toe temperature measurement at 12 h showed good ability to predict mortality (AUC [95% confidence interval): 0.72 [0.58–0.84]). At toe temperature ≤ 25.5 °C, the positive predictive value was 82% and the negative predictive value was 70% (Table 3). On the other hand, both serum lactate and CRT showed good ability to predict mortality at all timepoints (Table 3). The AUC for the serum lactate (AUC: 0.72, 0.78, and 0.81) and CRT (AUC: 0.74, 0.79, and 0.81) improved over the time points (Table 3). The best cut-off value for serum lactate was > 2.5–4.3 mmol/L with a positive predictive value of 84–100%. The best cut-off value for the CRT was > 3-4.2 s with a positive predictive value of 90–100% (Table 3).

**Table 3:** The AUC analysis for the ability to predict in-hospital mortality

	AUC (95%CI)	Sensitivity % (95% CI)	Specificity % (95% CI)	PPV % (95% CI)	NPV % (95% CI)	Cut-off value
Canthal-toe temperature gradient (°C)						
0	0.51 (0.37–0.64)	70 (54–83)	13 (2–41)	68 (52–82)	14 (2–43)	≤ 14.8
6 h	0.62 (0.48–0.75)	85 (70–94)	47 (21–73)	81 (65–91)	54 (25–81)	> 10.8
12 h	0.66 (0.52–0.78)	93 (80–98)	40 (16–68)	80 (66–91)	67 (30–93)	> 6.2
Room-toe temperature gradient (°C)						
0	0.58 (0.44–0.71)	69 (52–83)	53 (27–79)	79 (62–91)	40 (19–64)	> 0.5
6 h	0.57 (0.43–0.71)	67 (50–81)	60 (32–84)	81 (64–93)	41 (21–64)	>-0.6
12 h	0.62 (0.48–0.75)	97 (87–100)	33 (12–62)	79 (65–90)	83 (36–100)	>-10
Toe temperature (°C)						
0	0.51(0.37–0.65)	60 (43–75)	60 (32–84)	80 (61–92)	36 (18–58)	< 20.8
6 h	0.66(0.52–0.78)	85 (70–94)	53 (27–79)	83 (68–93)	57 (29–82)	≤ 23.3
12 h	0.72(0.58–0.84) *	92 (79–98)	47 (21–73)	82 (67–92)	70 (35–93)	≤ 25.5
Serum lactate (mmol/L)						
0	0.72(0.58–0.83) *	80 (64–91)	60 (32–84)	84 (69–94)	53 (28–77)	> 2.5
6 h	0.78(0.65–0.88) *	55 (39–70)	100 (78–100)	100 (85–100)	46 (28–64)	> 4.3
12 h	0.81(0.68–0.91) *	72 (55–85)	87 (60–98)	93 (78–99)	54 (33–74)	> 2.5
CRT (s)						
0	0.74(0.60–0.85) *	70 (54–83)	80 (52–96)	90 (74–98)	50 (29–71)	> 3
6 h	0.79(0.65–0.88) *	70 (54–83)	87 (60–98)	93 (78–99)	52 (31–72)	> 3
12 h	0.81(0.68–0.91)*	49 (32–65)	100 (78–100)	100 (82–100)	43 (26–61)	> 4.2
Clinical severity scores						
APACHE II score	0.75(0.61–0.86)*	62 (45–77)	77 (46–95)	89 (71–98)	40 (21–61)	> 18
SOFA score	0.77(0.64–0.88)*	46 (30–63)	92 (64–100)	945 (74–100)	36 (20–55)	> 8

APACHE II: acute physiology, and chronic health evaluation II score, AUC: area under receiver operating characteristic curve, CI: confidence interval, CRT: capillary refill time, PPV: positive predictive value, NPV: negative predictive value, SOFA: sequential organ failure assessment score. *Denotes statistical significance, *P* value < 0.05

The AUC for toe temperature at 12 h was comparable to that for the corresponding serum lactate and CRT (*P*-values: 0.185 and 0.346, respectively) as well as the APACHE II and SOFA scores (*P*-values: 0.975 and 0.798, respectively). The AUCs for serum lactate and CRT were comparable at all timepoints.

There was a significant negative correlation between toe temperature measurements and serum lactate (r [95%confidence interval]: -0.24 [-0.38 to -0.10]), while there was no correlation between each of the canthal-toe and room-toe temperature gradients and the serum lactate (Table 4).

**Table 4:** Correlation between temperature measurements and serum lactate

	Correlation Coefficient r (95% CI)	*P*-value
Canthal-toe temperature gradient	0.03 (-0.12 to 0.18)	0.688
Room-toe temperature gradient	0.003 (-0.15 to 0.16)	0.967
Toe temperature	-0.24 (-0.38 to -0.10)	0.002

CI: confidence interval

## Discussion

We evaluated the ability of central-toe temperature gradient and toe temperature, measured using infrared thermography, to predict outcomes of patients with septic shock. We found that non-survivors had lower toe temperature and higher canthal-toe temperature gradient 6 and 12 h after admission compared to survivors; however, the only temperature measurement that was able to predict patient’s outcome was the toe temperature 12 h after admission. Both serum lactate and CRT had good ability to predict in-hospital mortality at all time points of assessment. The ability of toe temperature to predict in-hospital mortality was comparable to that of serum lactate and CRT; however, the high positive predictive value for the serum lactate and CRT favors both measurements for ruling in in-hospital mortality, while the relatively high negative predictive value of toe temperature favors the later in ruling out in-hospital mortality.

Temperature measurements, in absolute values and gradients, had been considered accurate and non-invasive parameters for tissue perfusion. Evaluation of the perfusion of non-vital organs, such as skin temperature and CRT, is beneficial in patients with shock as these organs deteriorate earlier and recover later than vital organs [2]. However, the most common limitation of the use of skin temperature is the need to special probes [2]. The use of infrared thermal camera carries many advantages for being non-invasive and accurate [14]. Thermal camera is also economic as it does not require any consumables. Furthermore, thermal camera has the advantage of avoiding close contact with the patients which is warranted in the peri-pandemic era. Unlike our findings, a previous study by Amson et al. had reported good predictive properties for thermal imaging in predicting 8-day patient outcomes [16]. Several reasons might explain the difference between our study and Amson et al. study such as the different primary outcome. Amson et al. used eight-day mortality as endpoint in their study while we used in-hospital mortality. All participants in our study were postoperative admitted after emergency surgery while nearly 40% of the patients in Amson et al. study had non-surgical primary sources (lungs and urinary tract). In another study, Kimura et al. reported that skin temperature had a low performance for predicting major adverse events in children after cardiac surgery [17]; these findings come in line with our findings and support the assumption that skin temperature has a low-to-moderate accuracy as a perfusion index in post-surgical patients.

Patients with septic shock usually require surgical intervention to control the source of infection, and they are at high risk of mortality. The mortality rate in this study was within what was previously reported which can reach up to 76% [18]. Furthermore, our hospital, Cairo University hospital, is the largest tertiary hospital our country and one of the largest in Africa and the Middle East. Thus, most of the patients admitted to our emergency department are advanced and complicated cases and this might explain the high mortality rate in this group of patients. The complexity of the surgical status might not be adequately represented in the severity scoring systems which are calculated mainly from the vital signs and laboratory markers.

According to our results, serum lactate, CRT, SOFA, and APACHE II scores, measured as early as at the time of admission, can rule in patients with expected poor outcomes, while toe temperature after 12 h can rule out poor patient’s outcome. Thus, the use of thermal imaging could be combined with other variables to reach the best diagnostic accuracy. Most of the scoring systems have multiple variables which make them difficult to memorize and calculate at the bedside. Our study had some limitations such as being performed in one university hospital and strictly including emergency surgical patients. We limited the temperature recording to the first 12 h after admission. Therefore, future studies are warranted in different subgroups of surgical patients with longer period of assessment.

In conclusion, in post-operative emergency surgical patients with septic shock, high serum lactate and CRT can accurately predict in-hospital mortality and were superior to thermal imaging, especially in the positive predictive values. Toe temperature > 25.5 °C, measured using infrared thermal imaging, can exclude in-hospital mortality with a negative predictive value of 70%.

## Data Availability

The datasets used and/or analyzed during the current study are available from the corresponding author on reasonable request.
